# Intravenous ketamine for severe alcohol use disorder at Moi Teaching & Referral Hospital, Kenya: a case report

**DOI:** 10.1186/s13011-023-00519-0

**Published:** 2023-02-17

**Authors:** Florence Jaguga, Philip Kirwa, Benson Gakinya, Imran Manji, Thomas Andale, Daniel Kinyanjui, Edith Kamaru Kwobah, Felicita Mwangi, Kituyi Werunga, Josephat Kerema, Charles Kwobah, Eunice Temet, Julia Songok, Wilson K. Aruasa

**Affiliations:** 1 Moi Teaching and Referral Hospital, PO BOX -30100, Eldoret, Kenya; 2 School of Medicine, Moi University College of Health Sciences, PO BOX 4606, Eldoret, Kenya; 3 Albury Wodonga Health, Kerferd Unit 37/45 corner Dixon and docker streets, Wangaratta, Victoria 3677 Australia

**Keywords:** Ketamine, Off-label, Alcohol use disorder, Kenya, Case report

## Abstract

**Background:**

Alcohol use disorder is prevalent globally and in Kenya, and is associated with significant health and socio-economic consequences. Despite this, available pharmacological treatment options are limited. Recent evidence indicates that intravenous (IV) ketamine can be beneficial for the treatment of alcohol use disorder, but is yet to be approved for this indication. Further, little has been done to describe the use of IV ketamine for alcohol use disorder in Africa. The goal of this paper, is to: 1) describe the steps we took to obtain approval and prepare for off-label use of IV ketamine for patients with alcohol use disorder at the second largest hospital in Kenya, and 2) describe the presentation and outcomes of the first patient who received IV ketamine for severe alcohol use disorder at the hospital.

**Case presentation:**

In preparing for the off-label use of ketamine for alcohol use disorder, we brought together a multi-disciplinary team of clinicians including psychiatrists, pharmacists, ethicists, anesthetists, and members of the drug and therapeutics committee, to spearhead the process. The team developed a protocol for administering IV ketamine for alcohol use disorder that took into account ethical and safety issues. The national drug regulatory authority, the Pharmacy and Poison’s Board, reviewed and approved the protocol.

Our first patient was a 39-year-old African male with severe alcohol use disorder and comorbid tobacco use disorder and bipolar disorder. The patient had attended in-patient treatment for alcohol use disorder six times and each time had relapsed between one to four months after discharge. On two occasions, the patient had relapsed while on optimal doses of oral and implant naltrexone. The patient received IV ketamine infusion at a dose of 0.71 mg/kg. The patient relapsed within one week of receiving IV ketamine while on naltrexone, mood stabilizers, and nicotine replacement therapy.

**Discussion & conclusions:**

This case report describes for the first time the use of IV ketamine for alcohol use disorder in Africa. Findings will be useful in informing future research and in guiding other clinicians interested in administering IV ketamine for patients with alcohol use disorder.

## Background

Harmful alcohol use is associated with a significant health and socio-economic burden. In 2016, the harmful use of alcohol resulted in an estimated 3 million deaths (5.3% of all deaths) globally with most of those deaths arising from unintentional injuries (20.9%), digestive (21.3%), and cardiovascular diseases [1]. In the same year, harmful alcohol use further led to a large burden of disease and injury, causing 132.6 million Disability Adjusted Life Years (DALYs) which represented 5.1% of all DALYs in that year [1]. This burden is disproportionately heavy for the World Health Organization (WHO) Africa region [1]. In 2016, the aged-standardized alcohol-attributable burden of disease and injury was highest in the WHO African Region at 70.6 deaths and 3044 DALYs per 100 000 people [1].

The social and economic costs related to alcohol consumption are similarly substantial. In a systematic review and modeling study, that included studies from Africa, Europe, Asia, and North America, Manthey et al. 2021 [2], estimated the economic costs of alcohol consumption to amount to 1306 I$ per adult or 2.6% of the gross domestic product (GDP). The combined total tangible and intangible costs of alcohol harm to the economy were estimated at 10—12% of the 2009 GDP in South Africa [3]. At the family level, alcohol use contributes to interpersonal conflict, domestic violence, child abuse, and financial challenges [4], while within the workplace alcohol use has been linked to impaired work performance [5].

In Kenya, the prevalence of alcohol use disorder is 10% among 15–65-year-olds [6] and the total DALYS from alcohol use disorder [54,000] are among the highest in the continent [7]. A recent economic analysis found that the costs related to alcohol use disorder were US$ 34 million [8].

Despite the significant negative impact harmful alcohol consumption has on the economy and on health, available pharmacological treatment options remain limited. Currently, the only drugs for alcohol use disorder approved by major pharmaceutical regulatory bodies such as the Food and Drug Administration (FDA), and the European Medicines Agency (EMA), include naltrexone (oral and long-acting injection), disulfiram, acamprosate, gamma-hydroxybutyrate, and nalmefene. Of these, naltrexone, disulfiram, and acamprosate are found in the Kenyan market. However, naltrexone (tablet, injection, implant) is the only one that is listed as an essential drug by the Ministry of Health [9] and is therefore the only medication that is available within the public health sector.

Ketamine is a drug that has been approved both internationally and locally for use in anesthesia and procedural sedation [10]. It is an N-methyl-D-aspartate (NMDA) receptor antagonist. The NMDA receptor which is located in the brain plays a crucial role in learning, memory, and neuroplasticity. Due to these functions, the use of ketamine in mental health conditions has gained traction in recent times. The drug is efficacious in the management of depression [11, 12], suicidal ideation [13, 14] cocaine use disorder [15, 16], and heroin use disorders [17, 18]. There is growing evidence for the role of ketamine in the treatment of alcohol use disorder. Upregulation and altered NMDA function during chronic alcohol exposure is implicated in withdrawal and increased reactivity to alcohol cues, and cravings [19]. Ketamine is postulated to treat, alcohol use disorder by enhancing neuroplasticity and neurogenesis and blocking the reconsolidation of alcohol-related memories through its actions on the NMDA receptor [19].

In a randomized midazolam-controlled pilot trial conducted in the US, Dakwar et al. [20] compared the efficacy of a single ketamine infusion with midazolam. Participants (*N* = 40) had alcohol dependence with minimal psychiatric comorbidity. In that study, a single infusion of ketamine (a 2-min 0.11-mg/kg bolus in saline followed by a 50-min slow-drip intravenous infusion of 0.6 mg/kg) significantly increased the likelihood of abstinence, delayed the time to relapse, and reduced the likelihood of heavy drinking days compared with midazolam [20].

Das et al., [21] in a randomized controlled trial (RCT), investigated the efficacy of a combination of ketamine and retrieval of maladaptive alcohol memories in reducing motivation to drink and harmful drinking compared to ketamine only and retrieval only. The study was conducted among 90 adults from the United Kingdom (UK) with harmful/hazardous alcohol use. Patients had high levels of alcohol use (mean Alcohol Use Disorder Identification Test [AUDIT] scores of 22.13 ± 4.93). The authors found that intravenous (IV) ketamine (single infusion of 350 ng/ml for 30 min) following the brief retrieval of maladaptive cue-alcohol memories, produced a significantly greater reduction in the reinforcing effects of alcohol compared to retrieval only or ketamine only [21].

In a recent double-blind placebo-controlled phase 2 clinical trial, 96 patients with severe alcohol use disorder were randomly assigned to one of four conditions: 1) three weekly ketamine infusions (0.8 mg/kg IV over 40 min) plus psychotherapy, 2) three saline infusions plus psychotherapy, 3) three ketamine infusions plus alcohol education, or 4) three saline infusions plus alcohol education. In that study, there was a significantly greater number of days abstinent from alcohol in the ketamine group compared with the placebo group at the 6-month follow-up, with the greatest reduction in the ketamine plus therapy group compared with the saline plus education group [22].

Ketamine is affordable (a single 10 ml vial costs about US$ 4) and its use for procedural anesthesia is well established in level secondary and tertiary level health facilities within the Kenyan healthcare system. Moreover, the dosage of ketamine recommended for alcohol use disorder (0.8 mg/kg) [20–22] is much lower than that used for anesthesia (4.5 mg/kg) and therefore carries minimal risk. Ketamine is therefore an attractive option for alcohol use disorder management in Kenya. Ketamine is, however, yet to be approved for alcohol use disorder by major international pharmaceutical regulatory agencies such as the FDA and EMA. Similarly, ketamine has not been approved for this indication in Kenya by the Pharmacy and Poisons Board. The use of medication off-label may carry significant safety, and ethical risks but may be justified under specific circumstances provided the necessary regulatory, ethical, and clinical safeguards are put in place [23].

The goal of this paper is two-fold: 1) to describe the processes a team of clinicians at a tertiary level hospital in Kenya undertook in preparing to use IV ketamine off-label for alcohol use disorder and 2) to present a case of the first patient in our facility, who received IV ketamine for severe alcohol use disorder. Our paper aligns with the global strategy for harmful alcohol use which requires that adequate treatment services be provided for persons with alcohol use disorder [24].

### Setting

Moi Teaching and Referral Hospital is a multispecialty national teaching and referral hospital in Kenya [25]. The hospital serves the western half of Kenya, Eastern Uganda, Southern Sudan, Northern Tanzania, and the Democratic Republic of Congo amongst other areas. Altogether, the Hospital covers a population of over 25 million [25]. The facility has both outpatient and in-patient services that cater to patients with substance use disorders (SUDs). The number of patients seeking treatment for alcohol use disorder at the hospital has steadily grown over the years from about 400 patients per annum in 2017/2018 to 1500 per annum in 2021/2022. Treatment options for alcohol use disorder at the hospital include cognitive behavioral therapy (CBT) and naltrexone. The cost of naltrexone is prohibitive (US$120 for a month’s dose), and many patients are therefore unable to afford it. Moreover, many patients present with severe alcohol use disorder, and often relapse soon after completion of the in-patient or out-patient program. It is against this background that a team of clinicians from MTRH considered the use of IV ketamine off-label for severe alcohol use disorder within the facility.

### Preparing for use of IV ketamine for alcohol use disorder off-label

In July 2021, a multidisciplinary team of 12 hospital staff was constituted including hospital managers, researchers, psychiatrists, pharmacists, members of the drug and therapeutics committee, ethicists, and anesthetists. The team held a series of meetings during which deliberations were done on the need for introducing IV ketamine for severe alcohol use disorder at the hospital, and whether the available scientific evidence was adequate to justify the use of the medication off-label. The team additionally deliberated on the procedures and steps to be followed when administering the medication, and the necessary ethical, safety, and regulatory requirements to be met before using IV ketamine off-label. The team then developed a proposal for use of ketamine off-label within the hospital. The proposal included available literature on the efficacy of ketamine for severe alcohol use disorder, the safety profile of ketamine, and a proposed protocol for administering IV ketamine. The contents of the proposed protocol and the rationale for each component of the protocol have been outlined in Table 1.

**Table 1:** Summary of protocol for administering IV Ketamine for severe alcohol use disorder at Moi Teaching and Referral Hospital

Activity	Sub-activities	Rationale
**Review of each case by the Hospital Ethics Committee**	Determination by the Hospital Ethics Committee that the use of IV ketamine off-label is justified and adheres to ethical principles	To ensure that the benefits to the patient outweigh the risks and all available approved options have been tried unsuccessfully To ensure that all necessary information on risks and benefits will be given to the patient
**Review of each case by the Drug and Therapeutics Committee**	Determination by the Drug and Therapeutics Committee that it is safe to use IV ketamine for a particular patient	To ensure safety issues are addressed and to limit the occurrence of adverse events
**Consenting procedures**	Determination of decisional capacity using validated and standardized tools	To ensure that the patient has the mental capacity to understand the risks and benefits of the intervention and can give informed consent
	Obtain written consent from the patient after a detailed discussion of the risks and benefits of the treatment	To ensure that the patient voluntarily agrees to undergo treatment after an explanation of the risks and benefits
	Psychoeducation to family and Obtaining concurrence of family or other significant Next of Kin	To ensure that important support systems for the patient understand the goal of the intervention and are willing to support the patient
**Patient preparation**	Perform the following laboratory tests: complete blood count, liver function tests, renal function tests, random blood sugar	To limit the occurrence of adverse events and to allow for adverse events monitoring. Ketamine use may result in adverse renal and hepatic events and may cause hypoglycemia [26, 27]
	Conduct a drug toxicology screen	To rule out ketamine abuse and avoid ketamine overdose which could result in serious adverse events such as cardiovascular and respiratory depression [28]
	Perform an electrocardiogram (EKG)	To allow for adverse events monitoring. Ketamine can cause conduction abnormalities and arrhythmias [26, 27]
	Perform a head computed tomography scan	To rule out head injury, often associated with heavy alcohol use or alcohol withdrawal
	Conduct substance use and other psychiatric assessments as indicated using standardized and locally validated assessments	To facilitate objective assessments of improvement in substance use and comorbid psychiatric symptoms
	Admission for procedure 1–2 days prior	To allow for the above investigations and preparations to be conducted
**Dosing**	Administer a single infusion of low-dose ketamine as follows: a 2-min 0.11-mg/kg bolus in saline followed by a 50-min slow-drip IV infusion of 0.6 mg/kg	The dosing schedule is informed by a study conducted by Dakwar et al. [20]
**Patient monitoring during and after infusion**	Monitor blood pressure, conduct cardiac monitoring (EKG), pulse oximetry, and assessment for level of consciousness Perform monitoring of the above parameters before infusion, periodically, or continuously throughout the infusion. Do not discontinue monitoring until any adverse effects (respiratory, cardiac, neurologic, or psychiatric symptoms) have resolved Ensure availability of pulse oximetry in the event of respiratory depression requiring an airway intervention to assure adequacy of respiration	We adapted these guidelines from the Pennsylvania Guidelines for Safe administration of Low-dose Ketamine [27]. The guidelines recommend that at the minimum, the patient monitoring requirements indicated here are fulfilled [27]
**Psychosocial support**	Offer psychosocial support pre and post administration including education to family	The use of medication off-label may be stressful and cause anxiety to patients and families. It is important to offer psychosocial support so that the emotional and mental health needs of patients and families are met throughout the process
**Follow-up**	Follow-up and repeat laboratory investigations and substance use and other psychiatric assessments (as indicated) at months 1,3 and 6	To monitor both the long and short-term outcomes of the intervention

The proposal was reviewed by hospital management and two hospital committees i.e., the Hospital Ethics Committee and the Drugs and Therapeutics Committee. The Hospital Ethics Committee is responsible for handling any ethical issues that may arise during patient care while the Drugs and Therapeutics Committee is responsible for promoting rational drug use within the hospital. Both committees as well as hospital management approved the proposal.

The proposal was then forwarded to the Pharmacy and Poisons Board in Kenya for final approval. The board, a drug regulatory authority in Kenya, is responsible for regulating the practice of pharmacy and the manufacture and trade of drugs and poisons [29]. In October 2022, the Board evaluated and approved our proposal in reference to the Pharmacy and Poisons Board guidelines for emergency use and compassionate use authorization of health products and health technologies [30]. The Pharmacy and Poisons Board is yet to publish guidelines for the off-label use of medication in Kenya.

In their approval letter, the Pharmacy and Poisons Board gave the following conditions: That written informed consent be obtained from the patient or legal guardians; That information on product accountability be reported; That all adverse events be documented; That all fatal cases be reported within 48 h; That a report on patient outcomes be submitted; That off label use of ketamine be well documented in a patient registry. The Pharmacy and Poisons Board clarified that the approval to use IV ketamine off-label was not a marketing authorization. Throughout the planning process, we continuously engaged with the patient and his family and informed them that the use of IV ketamine for severe alcohol use disorder was still at the experimental stage.

### Case presentation for our first patient

The patient was a 39-year-old male with a 10-year history of heavy alcohol use, i.e., daily use of 500-750mls of spirits. During these 10 years, he had experienced a loss of control over alcohol use, he used alcohol despite experiencing harmful health consequences such as acute pancreatitis and gastritis, and he often experienced withdrawal symptoms upon abstinence. He had a long-standing history of cigarette smoking that largely accompanied alcohol use. Since 2019, cigarette smoking had intensified and he used about 10 sticks per day. He had been admitted to a substance use in-patient facility 6 times between 2010 and 2021 (Table 2), but always reverted to pre-treatment patterns of alcohol and tobacco use within one to four months after discharge. In between periods of in-patient treatment, the patient had been admitted multiple times with alcohol withdrawal delirium often accompanied by serious medical complications such as pancreatitis, alcoholic hepatitis, or upper gastrointestinal bleeding. Following in-patient treatment out-patient follow-up was inconsistent, partly because services that offer out-patient SUD care are scarce in Kenya.

**Table 2:** Patient’s substance use treatment history and time to relapse

Year	Treatment	Time to relapse for both alcohol and tobacco use
2010	Admitted for inpatient rehabilitation^a^ for 3 months	2 months after discharge
2013	Admitted for inpatient rehabilitation^b^ for 3 months	1 month after discharge
2014	Admitted for inpatient rehabilitation^a^ for 3 months	3 months after discharge
2017	Admitted for inpatient rehabilitation^a^ for 3 months	4 months after discharge
April to July 2019	Admitted for inpatient rehabilitation^b^ for 3 months; attended out-patient follow-up for 2 months Received oral naltrexone 50 mg once daily for 5 months. After this, he had a naltrexone implant (765 mg) inserted because oral naltrexone was out of stock. He relapsed a week after the implant was inserted Received nicotine replacement therapy and bupropion 150 mg once daily for tobacco use disorder for 3 months	2 months after discharge with naltrexone implant in-situ
February to August 2021	Admitted for inpatient rehabilitation^b^ for 6 months Received naltrexone 50 mg once daily for 6 months Received nicotine replacement therapy for tobacco use disorder for 3 months. He did not receive bupropion because it was out of stock	2 weeks after in-patient treatment while on oral naltrexone A decision was made to try IV ketamine in light of recent evidence showing efficacy for severe alcohol use disorder

^a^treatment was conducted in a residential setting that offers therapy based on alcohol anonymous and peer recovery support strategies

^b^treatment was conducted in a setting offering medically managed intensive in-patient services

The patient was diagnosed with bipolar disorder in 2009 and had experienced numerous depressive and manic episodes since then. Adherence to mood stabilizers was erratic. For this patient, it was generally difficult to determine whether mood symptoms triggered alcohol use or vice versa.

The patient had a family history of heavy alcohol use i.e. two out of seven of his siblings. In addition, his mother and one male sibling had a history of mental illness (depression and psychotic disorder respectively). The patient was married to one wife and had two children. His pattern of alcohol use had considerably interfered with his functioning at work, his career progression, and his family life.

The decision to use IV ketamine off-label for this patient was based on the following reasons: i) The patient had severe alcohol use disorder with significant social and occupational dysfunction and available evidence suggested the efficacy of IV ketamine for severe alcohol use disorder [20–22]. ii) Several attempts at treatment had been made using a combination of medication and psychotherapy with minimal improvement. All available evidence-based treatment options had been tried i.e. naltrexone (oral and implant), cognitive behavioral therapy, and alcoholic anonymous; iii) The team believed that the potential benefits to the patient, i.e. reduced alcohol consumption, reduced craving, and improvements to the quality of life outweighed the minimal risk of apnea, and raised blood pressure associated with the use of low dose ketamine.

We administered IV ketamine to the patient on 17^th^ November 2021, three months after his 6-month rehabilitation program ended (see Table 2). Before the infusion, the patient was admitted to our hospital. At the time of admission, he was experiencing alcohol withdrawal symptoms and was in a manic episode. The patient underwent treatment for alcohol withdrawal with parenteral diazepam and thiamine. He additionally received nicotine replacement therapy, olanzapine 10 mg per day, and valproate 1 g per day for mood stabilization. He achieved resolution of manic and alcohol withdrawal symptoms after 12 days and was deemed ready for the ketamine infusion.

In administering ketamine, we adhered to the protocol that had been prepared by the multi-disciplinary team above (Table 1). First, we sought and obtained approvals from hospital management, the Hospital Ethics Committee, the Drug and Therapeutics Committee, and the National Pharmacy and Poison Board. Next, we conducted a psycho-education session with both the wife and the patient. During the session, we shared with them information on the treatment and responded to any questions they had. We then assessed for decisional capacity using the Mini-Mental State Examination. The patient had a score of 29 and was determined to have adequate capacity to consent. We sought and obtained written informed consent from the patient and obtained the concurrence of the patient's wife.

We conducted all the necessary investigations per our protocol. The liver function tests i.e. aspartate transaminase (AST), alanine transaminase (ALT), alkaline phosphatase (ALP), bilirubin, and total protein were within normal levels. Gamma-glutamyl transferase (GGT) levels were marginally elevated at 75.0U/L (normal range 10.0–66.0U/L). All parameters in the complete blood count and renal function tests were within normal. A urine drug toxicology test was negative, an Electrocardiogram (EKG) was normal, and a head computed tomography (CT scan) showed brain atrophy.

The Alcohol Smoking and Substance Involvement Screening Test (ASSIST) scores were 36 for alcohol and 32 for tobacco and this was indicative of high-risk alcohol and tobacco use. We further assessed for disability using the World Health Organization- Disability Assessment Schedule (WHO-DAS) version 2.0. The findings of that assessment have been summarized in Table 3 below.

**Table 3:** Findings of assessment using the WHO-DAS 2.0

Domain	Mean score	Level of severity
Understanding and communicating	2.2	Mild
Getting around	1.8	Mild
Self-care	2.0	Mild
Getting along with people	2.8	Moderate
Life activities	2.5	Moderate
Participation in the society	3.5	Severe
Number of days difficulties were present in the past 30 days	21 days	

The patient received a single infusion of low-dose IV ketamine as follows: 10 mg bolus followed by a 50 mg slow-drip in saline infusion over 50 min per protocol. During infusion, we continuously conducted monitoring for cardiac functions (EKG), blood pressure, oxygen saturation levels monitoring, the level of consciousness, and the occurrence of psychiatric symptoms. We did not observe any adverse effects (respiratory, cardiac, neurologic, or psychiatric symptoms) during infusion and the vital signs remained within normal.

Ketamine was administered by an anesthetist in the high-dependency unit where there was availability of facilities for monitoring and supporting respiration in the event of respiratory depression requiring airway intervention.

Following the infusion, the patient remained in the hospital for 48 h. During this time, he was monitored for any adverse events related to ketamine use, and for the re-emergence of mood or psychotic symptoms. We additionally conducted a session of cognitive behavioral therapy after the infusion. No adverse events were noted in the 48 h after infusion and the patient was discharged home. One week after the infusion, the patient relapsed to prior patterns of alcohol and tobacco use.

## Discussion and conclusions

To the best of our knowledge, this is the first paper to describe the use of IV ketamine for alcohol use disorder in Africa. Prior literature on the subject has mostly originated from the western world [31].

The need to use ketamine off-label for alcohol use disorder at our facility brings to the fore the challenge of limited medication options for the management of alcohol use disorder in Kenya. Currently, naltrexone is the only medication for alcohol use disorder that has been listed as an essential medication and is therefore the only one that is available within the public health sector. For our patient, we did not try acamprosate or disulfiram because both were costly and were only available within the private health sector. We call for the listing of additional drugs such as acamprosate and disulfiram as essential medicines, to enhance the availability of treatment options for alcohol use disorder in Kenya.

Our patient relapsed back to pre-treatment levels of alcohol use soon after the IV ketamine infusion. Possible reasons include the intractable and severe nature of alcohol use disorder in our patient, the co-occurrence of psychiatric morbidities, and the ketamine dosing schedule that we used. While drug-drug interactions are a potential reason for the poor response to ketamine, we found no evidence suggesting possible interactions between ketamine and the medication (nicotine replacement therapy, olanzapine, and valproate) the patient was on [10]. Finally, the patient had significant distress as a result of socio-occupational dysfunction emanating from the alcohol use disorder and this may have contributed to the relapse.

Available studies do not provide adequate information on the type of patient for whom ketamine can be beneficial. Moreover, the impact of comorbidities on response to ketamine, as well as the optimal dosage and dosing frequency, are yet to be determined. Randomized Controlled Trials showing efficacy have used dosing frequencies ranging from 1–3 and doses ranging from 0.7 to 0.8 mg/kg. We call for more research in the area of ketamine for alcohol use disorder to address the question of optimal dosing schedule, and the impact of comorbidities and severity of alcohol use disorder on clinical outcomes following ketamine administration. We additionally call for research with diverse populations including people of African descent. Studies done so far using ketamine have focused on Caucasian populations [20–22] and findings may not be generalizable to African people.

Our patient relapsed while on optimal doses of naltrexone. This is not surprising and several studies have reported a lack of efficacy with approved drugs like naltrexone [31–33] and acamprosate [34]. In a meta-analysis conducted by Palpacuer et al., [34], no efficacy was observed for naltrexone and acamprosate for the management of alcohol use disorder. Available evidence further suggests that outcomes with naltrexone may be poor for African American populations [32, 35]. Given the magnitude of health and socio-economic harm associated with alcohol use disorder, there is a need to increase efforts aimed at developing and testing more effective pharmacological treatments for alcohol use disorder. Several randomized controlled trials aimed at testing the efficacy of pharmacotherapies for alcohol use disorder have been conducted. Drugs that have been tested include topiramate [36], zonisamide [37], gabapentin [38], ondansetron [39], memantine [40], and sodium oxybate [41] among others. None of these trials have however been conducted in Africa. We call for more research focusing on pharmacotherapies for alcohol use disorder in Africa.

Overall, we faced no noteworthy challenges during the preparatory processes and the actual administration of the IV ketamine. We discovered that there were no guidelines for the off-label use of medication in Kenya and we call on the relevant stakeholders to develop them. Our patient did not experience any adverse events throughout infusion and post-infusion. This is consistent with observations in existing trials of IV ketamine which reported that side effects were minimal and that the low-dose ketamine was well tolerated [20–22].

Limitations to this report are that it is a single case report and cannot provide conclusive evidence against the efficacy of IV ketamine for alcohol use disorder. Nonetheless, it provides for the first time real-world information that could guide clinical practice and the design of future trials.

This case report provides information that could be beneficial for stakeholders planning for off-label use of IV ketamine for alcohol use disorder both in Kenya and globally. Secondly, this case report provides information that could guide the design of future trials aimed at exploring the efficacy of IV ketamine for alcohol use disorder.

As a next step, the authors plan to refine the ketamine protocol to include the three weekly infusion regimen as delivered in the most recent clinical trial (a phase 2 double-blind placebo-controlled trial) [22] instead of the single infusion described in this case report. Secondly, the authors plan to conduct a randomized control trial to examine the efficacy of IV ketamine among Kenyan patients with alcohol use disorder.

## Data Availability

Not applicable.

## Abbreviations

ALT: Alanine transaminase
AST: Aspartate transaminase
AUDIT: Alcohol Use Disorder Identification Test
CBT: Cognitive Behavioral Therapy
DALY: Disability Adjusted Life Year
EKG: Electrocardiogram
EMA: European Medicines Agency
GDP: Gross Domestic Product
GGT: Gammaglutamyl transferase
I$: International Dollar
IV: Intravenous
FDA: Food and Drug Administration
g: Grams
mg: Milligrams
NMDA: N-methyl-D-aspartate
NHIF: National Hospital Insurance Fund
RCT: Randomized Controlled Trial
SDG: Sustainable Development Goals
SUD: Substance use disorder
UK: United Kingdom
US$: United States Dollar
US: United States
WHO-DAS: World Health Organization-Disability Assessment Schedule
WHO: World Health Organization
